# Sex-Specific Association Between XPC rs2228001 Polymorphism and Parkinson’s Disease Risk in a Mexican Population: A Case–Control Study Exploring Gene–Environment Interactions

**DOI:** 10.3390/brainsci15091008

**Published:** 2025-09-18

**Authors:** Karla Mariana Alvarado-Retana, Daniel Francisco Ramos-Rosales, Elizabeth Irasema Antuna-Salcido, Sergio Manuel Salas-Pacheco, Francisco Xavier Castellanos-Juárez, Edna Madai Méndez-Hernández, Alma Cristina Salas-Leal, Osmel La Llave-León, Gerardo Quiñones-Canales, Oscar Arias-Carrión, Ada Sandoval-Carrillo, José Manuel Salas-Pacheco

**Affiliations:** 1 Instituto de Investigación Científica, Universidad Juárez del Estado de Durango, Durango 34000, Mexico; mariana.alvaret@gmail.com (K.M.A.-R.); daniel.ramos@ujed.mx (D.F.R.-R.); elizabeth.antuna@ujed.mx (E.I.A.-S.); xavier_castellanos@hotmail.com (F.X.C.-J.); edna_madai@hotmail.com (E.M.M.-H.); alma.salas@ujed.mx (A.C.S.-L.); olallavel@ujed.mx (O.L.L.-L.); adda-sandoval@hotmail.com (A.S.-C.); 2 Facultad de Odontología, Universidad Juárez del Estado de Durango, Durango 34070, Mexico; phc.smsp@gmail.com; 3 Hospital Santiago Ramón y Cajal-ISSSTE, Durango 34070, Mexico; gqc55@hotmail.com; 4 División de Neurociencias, Clínica, Instituto Nacional de Rehabilitación Luis Guillermo Ibarra Ibarra, Ciudad de México 14389, Mexico; ariasemc2@gmail.com; 5 Tecnologico de Monterrey, Escuela de Medicina y Ciencias de la Salud, Mexico City 14380, Mexico

**Keywords:** Parkinson’s disease, nucleotide excision repair, *XPC*, rs2228001

## Abstract

Background/Objectives: Emerging evidence implicates impaired DNA repair mechanisms in the pathogenesis of Parkinson’s disease (PD), particularly in the context of oxidative stress and environmental exposures. This study investigated the association between five polymorphisms in nucleotide excision repair (NER) pathway genes and PD susceptibility in a northern Mexican mestizo population. Methods: We conducted a case–control study including 137 patients with clinically diagnosed PD and 137 age- and sex-matched controls. Genomic DNA was isolated from peripheral blood, and genotyping of *ERCC1* (rs11615), *ERCC2* (rs13181), *XPA* (rs1800975), *XPC* (rs2228001), and *XPF* (rs1799801) was performed using TaqMan real-time PCR assays. Associations between genotype frequencies and PD were evaluated using logistic regression models adjusted for age, sex, and pesticide exposure. Results: A significantly higher prevalence of pesticide exposure was observed in PD patients than in controls (OR 2.08, 95% CI 1.18–3.68; *p* = 0.01). The *XPC* rs2228001 C/C genotype was independently associated with increased PD risk in males (OR 3.25, 95% CI 1.07–9.85; *p* = 0.042), even after adjusting for uric acid, pesticide exposure, and cognitive status (MMSE score). No significant associations were found for other NER-related polymorphisms. Male PD patients also exhibited significantly lower serum uric acid levels than controls (*p* = 0.046), supporting a link between oxidative stress and disease vulnerability. Conclusions: Our findings suggest a sex-specific genetic contribution to PD susceptibility involving the *XPC* rs2228001 variant, particularly in the context of pesticide exposure. These results underscore the relevance of DNA repair pathways in PD pathogenesis and highlight the importance of integrated models incorporating genetic and environmental risk factors.

## 1. Introduction

Parkinson’s disease (PD) is a progressive neurodegenerative disorder and the second most common cause of age-related disability worldwide, affecting more than 1% of individuals over the age of 65 years [1,2]. With rising life expectancy and aging populations, the global burden of PD is projected to double by 2030, representing a major public health and economic challenge [3]. Despite significant advances in symptomatic therapies, PD remains incurable, and disease-modifying interventions are still lacking [4,5].

The neuropathological hallmark of PD is the selective degeneration of dopaminergic neurons in the substantia nigra pars compacta, leading to striatal dopamine deficiency and the emergence of cardinal motor symptoms, including resting tremor, rigidity, bradykinesia, and postural instability [1,2]. However, non-motor features—such as cognitive decline, depression, sleep-related disorders and autonomic dysfunction—are now recognized as integral to disease progression and quality of life [6,7]. Nonetheless, certain non-motor symptoms, particularly sleep disturbances, may also occur in atypical parkinsonian syndromes such as PSP and CBS, underscoring the need for careful differential diagnosis to prevent misclassification [8].

Although the pathogenesis of PD is multifactorial, emerging evidence highlights the role of genomic instability and impaired DNA repair mechanisms in neuronal vulnerability [9]. Neurons are particularly susceptible to DNA damage due to high metabolic demand, sustained oxidative stress, and limited regenerative capacity [10]. In this context, neuroinflammation—particularly microglial activation—has been implicated as a key driver of PD pathogenesis, promoting chronic oxidative stress and neurotoxicity through the release of pro-inflammatory cytokines and reactive oxygen species (ROS) [11,12]. Mitochondrial dysfunction further exacerbates ROS production, amplifying oxidative DNA damage in postmitotic neurons [13]. These mechanisms support the hypothesis that impaired DNA repair—especially in response to oxidative and environmental insults—may play a central role in dopaminergic neuron degeneration [14].

Supporting this hypothesis, recent studies have provided direct molecular and pathological evidence implicating DNA damage and repair failure in the progression of PD. A longitudinal analysis from the Parkinson’s Progression Markers Initiative (PPMI) identified a peripheral DNA damage signature (marked by transcriptional repression of long neuronal genes) that not only correlated with accelerated motor decline but was also recapitulated in dopaminergic neurons of the substantia nigra in PD patients [15]. Complementing these findings, oxidative lesions to cytoplasmic DNA and RNA have been detected in substantia nigra neurons, offering histopathological confirmation of nucleic acid damage in affected brain regions [16]. Additionally, broader studies of neurodegeneration have revealed that deficiencies in key DNA repair pathways, particularly those countering oxidative stress, contribute significantly to neuronal dysfunction and loss [17]. Together, these converging lines of evidence reinforce the emerging paradigm that DNA repair dysfunction is not merely a downstream consequence but a potential upstream driver of neurodegeneration in PD.

Among the DNA repair systems, base excision repair (BER) has long been implicated in the response to oxidative DNA damage. However, recent studies suggest that the nucleotide excision repair (NER) pathway—traditionally associated with the removal of bulky DNA adducts and helix-distorting lesions—may also play a critical role in maintaining genomic integrity in postmitotic neurons [18,19].

The NER pathway involves several key proteins, including ERCC1, ERCC2 (also known as XPD), XPA, XPC, and XPF, which coordinate damage recognition, DNA unwinding, and excision of damaged strands. Functional variants in genes encoding these proteins have been previously associated with increased cancer risk and defective repair activity, but their role in neurodegenerative disorders remains underexplored. Recent reports suggest that polymorphisms in NER-related genes may modulate neuronal resilience to oxidative stress and environmental insults such as pesticide exposure, both of which are established contributors to PD risk [20,21].

Heritability estimates from twin and family studies indicate that approximately 30% of PD susceptibility can be attributed to genetic factors [3]. However, most known risk loci do not fully account for disease variability, particularly in underrepresented populations. This highlights the need to identify additional genetic contributors, especially those involved in molecular pathways relevant to neurodegeneration.

In this context, we investigated the association between PD and five single-nucleotide polymorphisms (SNPs) located in NER pathway genes—*ERCC1* (rs11615), *ERCC2* (rs13181), *XPA* (rs1800975), XPC (rs2228001), and XPF (rs1799801)—in a northern Mexican mestizo population. This study aimed to elucidate the potential contribution of DNA repair capacity to PD susceptibility and to explore gene–environment interactions in a genetically and environmentally distinct cohort.

## 2. Materials and Methods

### 2.1. Study Population and Ethical Considerations

This case–control study included 274 participants, consisting of 137 patients diagnosed with idiopathic Parkinson’s disease (PD) and 137 age- and sex-matched healthy controls without a history of neurodegenerative disorders. Participants were consecutively recruited from the Neurology Department of Hospital Doctor Santiago Ramón y Cajal, Instituto de Seguridad y Servicios Sociales de los Trabajadores del Estado (ISSSTE), located in Durango, Mexico.

Board-certified neurologists established PD diagnoses according to the UK Parkinson’s Disease Society Brain Bank clinical diagnostic criteria. Control participants were selected from the same geographical area and confirmed to be free of neurological or psychiatric diagnoses through clinical interview and review of medical records.

The study protocol received ethical approval from the institutional review board of the participating hospital. All procedures adhered to the principles of the Declaration of Helsinki. Written informed consent was obtained from each participant before inclusion. To ensure data privacy, clinical and genetic data were anonymized and stored in a secure database accessible only to the study investigators.

### 2.2. Clinical Evaluation of Motor and Non-Motor Symptoms

Motor and non-motor features of Parkinson’s disease were systematically assessed using standardized clinical tools. Global disease severity was evaluated with the Unified Parkinson’s Disease Rating Scale (UPDRS), which encompasses four subscales: mentation, behavior, and mood (Part I); activities of daily living (Part II); motor examination (Part III); and motor complications (Part IV). Only Part III (motor) scores were used in statistical analysis due to clinical relevance and availability. In addition, global cognitive status was evaluated with the Mini-Mental State Examination (MMSE), providing a standardized measure of cognitive function across domains such as orientation, attention, memory, and language.

Additional variables—including age, sex, serum glucose, uric acid, exposure to heavy metals, and self-reported exposure to pesticides—were collected through standardized interviews and confirmed with laboratory data when applicable. Pesticide exposure was recorded as a binary variable (Yes/No) based on occupational or residential proximity to agricultural areas.

### 2.3. Genomic DNA Extraction and SNP Genotyping

Peripheral venous blood samples (5 mL) were collected in EDTA tubes and processed within 2 h of collection. Genomic DNA was extracted using a modified salting-out method, as described by Bartlett and White [22]. DNA concentration and purity were quantified using a NanoDrop™ 2000 spectrophotometer (Thermo Fisher Scientific, Waltham, MA, USA), with A260/A280 ratios of 1.8–2.0 considered acceptable.

Five single-nucleotide polymorphisms (SNPs) in NER pathway genes were selected based on prior evidence of functional relevance and allele frequency in Latin American populations. Genotyping was performed using TaqMan^®^ SNP Genotyping Assays (Applied Biosystems, Foster City, CA, USA) on a StepOne™ Real-Time PCR System, following the manufacturer’s instructions.

Each 10 µL PCR reaction contained:

- 10 ng of genomic DNA.
- 0.625 µL of allele-specific TaqMan probe (assay ID listed below).
- 5.0 µL of TaqMan Genotyping Master Mix.

Furthermore, nuclease-free water was used to reach the final volume. The thermal cycling protocol consisted of an initial denaturation at 95 °C for 10 min, followed by 40 amplification cycles of 95 °C for 15 s and 60 °C for 1 min. Genotype calls were made using StepOne Software v2.3 with automatic allele discrimination. No-call rates were <5% across all assays.

Assay identifiers:

- *ERCC1* rs11615–C_2532959_20.
- *ERCC2* rs13181–C_3145033_10.
- *XPA* rs1800975–C_482935_1_.
- *XPC* rs2228001–C_234284_1.
- *XPF* rs1799801–C_7487514_10.

Negative controls (no DNA template) and positive controls of known genotype were included on each plate to ensure assay fidelity.

### 2.4. Statistical Analysis

Statistical analysis was conducted using SPSS version 22.0 (IBM Corp., Armonk, NY, USA) and the web-based SNPStats tool (https://www.snpstats.net, accessed on 6 June 2025). Analyses adhered to the STREGA (Strengthening the Reporting of Genetic Association Studies) guidelines to ensure transparency and reproducibility.

#### 2.4.1. Data Preprocessing

- Continuous variables were assessed for normality using the Kolmogorov–Smirnov test.
- Variables with normal distribution are summarized as mean ± standard deviation (SD) and were compared using two-tailed independent-samples *t*-tests.
- Non-normally distributed data are reported as median (IQR) and were compared using the Mann–Whitney U test.
- Categorical variables are expressed as frequencies and proportions and were compared using Pearson’s chi-square (χ^2^) test.

#### 2.4.2. Genetic Association Testing

- Allele frequencies were compared between groups using the χ^2^ test, and genotype distributions were tested for Hardy–Weinberg equilibrium (HWE) in controls.
- Logistic regression models were applied to estimate the odds ratios (ORs) and 95% confidence intervals (CIs) for the association between each SNP and PD risk.
- Models were adjusted for pesticide exposure and uric acid where applicable.

Stratified analysis was conducted to examine sex-specific genetic effects. Haplotype analysis for ERCC1 rs11615 and *ERCC2* rs13181 was performed using SNPStats to assess combinatorial effects on PD risk. The most frequent haplotype was used as the reference, and ORs with 95% CIs were reported. A two-sided *p*-value < 0.05 was considered statistically significant throughout. No corrections were applied for multiple comparisons due to the exploratory nature of this study.

## 3. Results

### 3.1. Clinical and Sociodemographic Characteristics of Participants

The study enrolled 274 individuals, including 137 patients with clinically diagnosed PD and 137 age- and sex-matched controls without neurological disease. The distribution of males and females was identical in both groups (73/64), and mean ages were closely matched, indicating adequate demographic comparability. PD patients showed significantly lower MMSE scores than controls (25.95 ± 4.69 vs. 27.68 ± 4.59; *p* = 0.002), indicating the presence of cognitive impairment in the PD group (Table 1).

Notably, exposure to pesticides was significantly more frequent among individuals with PD than among controls (30.65% vs. 17.51%; *p* = 0.011), with a corresponding odds ratio (OR) of 2.08 (95% CI 1.18–3.68), suggesting a potential environmental risk factor. Among male participants, the difference in exposure remained significant (*p* = 0.012), reinforcing previous findings on sex-specific environmental vulnerabilities.

Biochemical profiling revealed a significant reduction in serum uric acid concentrations in male patients with PD compared with male controls (5.60 ± 1.47 vs. 6.07 ± 1.34 mg/dL; *p* = 0.047; Table 2). These findings support the hypothesis that lower uric acid levels—possibly reflecting diminished antioxidant capacity—may be linked to increased neurodegenerative risk. Regarding cognitive performance, male and female PD patients scored significantly lower on the Mini-Mental State Examination (MMSE) compared to controls (*p* = 0.031), indicating mild cognitive impairment associated with the disease.

### 3.2. Association of NER Gene Polymorphisms with PD Susceptibility

Five single-nucleotide polymorphisms (SNPs) within genes of the nucleotide excision repair (NER) pathway were evaluated for their association with PD: *ERCC1* (rs11615), *ERCC2* (rs13181), *XPA* (rs1800975), *XPC* (rs2228001), and *XPF* (rs1799801). All genotype distributions conformed to the Hardy–Weinberg equilibrium.

Among these, a statistically significant difference was detected in allele distribution for *XPC* rs2228001. The C allele was more frequent in PD cases than in controls (36% vs. 27%; *p* = 0.02; Table 3). Although individuals homozygous for the C allele (C/C genotype) showed a higher risk of PD after adjusting for pesticide exposure and cognitive status (MMSE score), the association did not reach statistical significance (OR 2.35, 95% CI 1.08–5.11; *p* = 0.087; Table 4).

To further explore whether pesticide exposure modified the genotype distribution within PD patients, we performed a stratified analysis (Supplementary Table S1). No significant associations were observed between *XPC* genotypes and pesticide exposure status among PD cases, although the frequency of the C/C genotype was slightly lower in exposed individuals (11.9%) compared to those unexposed (20.0%) (OR 0.46, 95% CI 0.15–1.39; *p* = 0.15). This finding suggests that the increased PD risk associated with the C/C genotype may not be driven solely by pesticide exposure.

By contrast, no significant differences in allele or genotype frequencies were observed for *ERCC1* rs11615, *ERCC2* rs13181, *XPA* rs1800975, or *XPF* rs1799801, suggesting that these variants may not contribute meaningfully to PD risk in this population (Table 3 and Table 4).

A sex-stratified association analysis of the NER pathway SNPs revealed that, after adjusting for uric acid and pesticide exposure, male carriers of the C/C genotype exhibited a significantly increased risk of PD (OR 3.25, 95% CI 1.07–9.85; *p* = 0.042; Table 5). This pattern was not observed in females (Table 6), suggesting a possible interaction between sex-specific biological or environmental factors and genetic susceptibility.

### 3.3. Haplotype Analysis of ERCC1 and ERCC2

Given their proximity on chromosome 19 and shared involvement in the NER pathway, a haplotype analysis was conducted to assess the combined influence of *ERCC1* rs11615 and *ERCC2* rs13181 on PD susceptibility. Four haplotypes were identified (C–A, T–A, C–C, T–C), none of which were significantly associated with altered PD risk (Table 7). The most common haplotype, C–A, was used as the reference. While the T–A haplotype showed a modest trend toward increased risk (OR 1.12, 95% CI 0.70–1.81), the association did not reach statistical significance (*p* = 0.59). These findings suggest limited combinatorial effects of these two SNPs in this cohort.

## 4. Discussion

This study provides new evidence linking environmental and genetic risk factors to PD susceptibility, with particular emphasis on male-specific vulnerability. We observed a significantly higher frequency of self-reported pesticide exposure in patients with PD compared with matched controls, a finding that persisted in male participants. These results are consistent with those of prior research implicating environmental toxins—particularly pesticides—as contributors to dopaminergic neurodegeneration through mechanisms involving oxidative stress, mitochondrial dysfunction, and inhibition of dopamine metabolism [23,24,25,26]. The sex-specific pattern echoes earlier findings suggesting that occupational exposure and behavioral risk profiles may modulate susceptibility in men [27].

Importantly, even after adjustment for pesticide exposure, we identified a significant association between the *XPC* rs2228001 C/C genotype and increased risk of PD in male participants. This association supports the hypothesis that genetic variability in DNA repair pathways may act independently or synergistically with environmental insults to influence disease onset [28]. Our results add to a growing body of literature suggesting that impaired genomic maintenance contributes to neuronal vulnerability in neurodegenerative diseases [9].

In addition, we found that male PD patients had significantly lower serum uric acid levels compared with controls, consistent with previous studies [29,30]. Uric acid has been proposed as an endogenous antioxidant capable of scavenging reactive oxygen species, and lower levels may reflect a reduced capacity to buffer oxidative stress—a key mechanism implicated in PD pathogenesis [28]. While not a primary endpoint, this metabolic difference reinforces the view that systemic redox imbalance may interact with genetic susceptibility.

PD patients exhibited significantly lower MMSE scores compared to healthy controls (25.95 ± 4.69 vs. 27.68 ± 4.59; *p* = 0.002), reinforcing the well-established notion that cognitive impairment is a core non-motor feature of Parkinson’s disease. This observation was consistent across both sexes, as male and female PD patients demonstrated significantly lower MMSE performance relative to sex-matched controls (*p* = 0.031), supporting the hypothesis that cognitive decline in PD is not strictly sex-dependent, but rather an intrinsic component of disease progression. These findings are in line with those of previous reports indicating that 25–30% of newly diagnosed PD patients present with mild cognitive impairment, which in many cases progresses to Parkinson’s disease dementia [31,32]. The observed reduction in MMSE scores likely reflects early dysfunction in frontostriatal and temporoparietal circuits, potentially driven by dopaminergic and cholinergic deficits, as well as broader neurodegenerative processes extending beyond the nigrostriatal pathway [33,34].

The integrity of neuronal DNA is essential for the long-term survival of postmitotic cells. While the base excision repair (BER) pathway has traditionally been emphasized in PD, emerging evidence suggests a role for the nucleotide excision repair (NER) system—particularly in response to helix-distorting lesions induced by environmental exposures [18,19]. Among the five polymorphisms studied in NER-related genes (*ERCC1*, *ERCC2*, *XPA*, *XPC*, and *XPF*), only *XPC* rs2228001 showed a significant association with PD. The variant results in a Lys939Gln substitution and has previously been linked to reduced repair capacity and increased cancer risk, including cervical, bladder, prostate, and colorectal cancers [25,35,36,37]. Functional data suggest that the C/C genotype may impair lesion recognition or recruitment of downstream repair factors [37], and studies in agricultural workers exposed to pesticides have reported increased DNA damage in *XPC* C/C carriers [38]. Taken together, these findings support a plausible biological mechanism by which compromised DNA repair may sensitize dopaminergic neurons to environmental stressors, thereby increasing PD risk.

In contrast, no significant associations were identified for the other four SNPs examined. Although this does not preclude a role for ERCC1, ERCC2, XPA, or XPF in PD pathogenesis, the functional impact of the specific polymorphisms selected in this study may be limited. Alternatively, their effects may be modulated by population-specific genetic backgrounds or environmental exposures not captured in our analysis.

Several limitations should be acknowledged. First, the sample size—while adequately powered for moderate associations—may lack sensitivity to detect smaller genetic effects or complex gene–environment interactions. Second, pesticide exposure was assessed via self-report and lacked granularity regarding chemical classes, duration, and intensity of exposure, potentially introducing recall bias. Third, recruitment from a single center (ISSSTE General Hospital in Durango) may introduce selection bias and limit sample representativeness. Although the hospital serves a broad catchment area in northern Mexico, findings may not fully generalize to PD populations in other healthcare settings or regions. Fourth, although PD encompasses clinically heterogeneous subtypes (e.g., tremor-dominant, akinetic-rigid, or early-onset forms), our study did not stratify participants by clinical phenotype. This limits the ability to determine whether the observed association with the *XPC* rs2228001 variant is subtype-specific. Future studies should incorporate this stratification to better delineate gene–phenotype relationships. Fifth, while we followed STREGA guidelines, we did not apply corrections for multiple comparisons due to the exploratory nature of the study. This decision was consistent with similar genetic association reports and aimed to avoid overly conservative adjustments that could increase the risk of Type II error given the moderate sample size. Moreover, we did not perform functional validation to assess the biological consequences of the *XPC* rs2228001 variant on DNA repair activity in neuronal models. Finally, replication in independent cohorts is essential before these findings can be translated into clinical risk stratification tools.

Despite these limitations, our study identifies *XPC* rs2228001 C/C as a potential genetic risk factor for PD in men and reinforces the importance of considering DNA repair capacity in the broader landscape of PD pathogenesis. These findings advocate for a paradigm shift in the way we understand neurodegeneration—not only as a consequence of protein misfolding and synaptic failure but also as a process potentially driven by cumulative genomic instability. This opens new avenues for research into DNA repair modulation as a therapeutic strategy.

Future investigations should aim to replicate these associations in larger, multiethnic cohorts and explore the functional impact of the *XPC* variant using in vitro neuronal models. Experimental approaches may include assessing DNA damage response under oxidative stress, evaluating NER pathway efficiency, and characterizing downstream transcriptional changes. Such work is essential to determine whether impaired repair contributes causally to PD pathophysiology or reflects an epiphenomenon of broader cellular dysfunction.

## 5. Conclusions

This study provides evidence for a significant association between pesticide exposure and increased risk of Parkinson’s disease, particularly among male individuals. Additionally, we report that the *XPC* rs2228001 C/C genotype is independently associated with PD susceptibility in men, even after adjustment for environmental exposures. No associations were observed for other NER-related polymorphisms. Our findings underscore the relevance of DNA repair mechanisms in the pathogenesis of PD and highlight the need for integrative models that account for both genetic and environmental contributions. Further research—including replication studies and functional analyses—is warranted to validate these associations and clarify their mechanistic role in neurodegeneration.

## Figures and Tables

**Table 1:** Clinical characteristics of the study population.

Variable	Controls (*n* = 137)	PD Cases (*n* = 137)	*p*-Value
Age (years)	70.28 ± 9.14	70.45 ± 9.07	0.87 ^1^
Sex (M/F)	73/64	73/64	1.00 ^+^
Glucose (mg/dL)	112.80 ± 31.61	110.63 ± 18.35	0.488 ^1^
Uric acid (mg/dL)	5.94 ± 1.41	5.65 ± 1.59	0.114 ^1^
Metal exposure (Yes/No)	24/113	29/108	0.444 ^+^
Pesticide exposure (Yes/No)	24/113	42/95	0.011 ^+^
MMSE	27.68 ± 4.59	25.95 ± 4.69	0.002 ^1^
UPDRS	—	62 (43–83.5)	—

Abbreviations: ^1^ = Student *t*-test; ^+^ = χ^2^; PD, Parkinson’s disease; MMSE = Mini-Mental State Examination; UPDRS = Unified Parkinson’s Disease Rating Scale.

**Table 2:** Clinical characteristics of participants stratified by sex.

Variable	Males			Females		
	Controls (*n* = 73)	PD Cases (*n* = 73)	*p*-Value	Controls (*n* = 64)	PD Cases (*n* = 64)	*p*-Value
Age (years)	70.45 ± 9.93	70.32 ± 9.27	0.938 ^1^	70.09 ± 8.22	70.60 ± 8.9	0.734 ^1^
Glucose (mg/dL)	114.36 ± 33.81	113.25 ± 17.15	0.804 ^1^	112.64 ± 26.18	107.68 ± 19.36	0.226 ^1^
Uric acid (mg/dL)	6.07 ± 1.34	5.60 ± 1.47	0.046 ^1^	5.78 ± 1.48	5.70 ± 1.72	0.77 ^1^
Metal exposure (Yes/No)	24/49	28/45	0.489 ^+^	0/64	1/63	
Pesticide exposure (Yes/No)	24/49	39/34	0.012 ^+^	0/64	3/61	0.080 ^+^
MMSE	27.43 ± 4.67	25.73 ± 4.72	0.031 ^1^	27.97 ± 4.51	26.19 ± 4.68	0.031 ^1^
UPDRS	—	64 (42–87.25)	—	—	62 (49.5–80)	

Abbreviations: ^1^ = Student *t*-test; ^+^ = χ^2^; PD, Parkinson’s disease; MMSE = Mini-Mental State Examination; UPDRS = Unified Parkinson’s Disease Rating Scale.

**Table 3:** Allele frequency distribution of NER gene polymorphisms.

Gene (SNP)	Controls (*n* = 274)	PD Cases (*n* = 274)	*p*-Value ^+^
*ERCC1* (rs11615)	C: 0.70 (191), T: 0.30 (83)	C: 0.67 (184), T: 0.33 (90)	0.51
*ERCC2* (rs13181)	A: 0.77 (211), C: 0.23 (63)	A: 0.81 (222), C: 0.19 (52)	0.24
*XPA* (rs1800975)	C: 0.71 (195), T: 0.29 (79)	C: 0.69 (190), T: 0.31 (84)	0.64
*XPC* (rs2228001)	A: 0.73 (201), C: 0.27 (73)	A: 0.64 (176), C: 0.36 (98)	0.02
*XPF* (rs1799801)	T: 0.74 (203), C: 0.26 (71)	T: 0.79 (217), C: 0.21 (57)	0.18

^+^ = χ^2^.

**Table 4:** Genotype frequency distribution and association with PD risk.

Gene (SNP)	Genotype	Controls (*n* = 137)	PD Cases (*n* = 137)	OR ^&^ (CI 95%)	*p*-Value ^+^
*ERCC1* (rs11615)	C/C	68 (49.6%)	59 (43.1%)	Ref	
	C/T	55 (40.1%)	66 (48.2%)	1.48 (0.88–2.48)	0.27
	T/T	14 (10.2%)	12 (8.8%)	0.92 (0.38–2.20)	
*ERCC2* (rs13181)	A/A	83 (60.6%)	88 (64.2%)	Ref	
	C/A	45 (32.9%)	45 (32.9%)	0.97 (0.57–1.64)	0.32
	C/C	9 (6.6%)	4 (2.9%)	0.40 (0.12–1.37)	
*XPA* (rs1800975)	C/C	81 (59.1%)	74 (54%)	Ref	
	C/T	33 (24.1%)	41 (29.9%)	1.22 (0.69–2.17)	0.79
	T/T	23 (16.8%)	22 (16.1%)	1.09 (0.55–2.15)	
*XPC* (rs2228001)	A/A	77 (56.2%)	63 (46%)	Ref	
	A/C	47 (34.3%)	50 (36.5%)	1.24 (0.72–2.12)	0.087
	C/C	13 (9.5%)	24 (17.5%)	2.35 (1.08–5.11)	
*XPF* (rs1799801)	T/T	77 (56.2%)	87 (63.5%)	Ref	
	C/T	49 (35.8%)	43 (31.4%)	0.82 (0.49–1.39)	0.48
	C/C	11 (8%)	7 (5.1%)	0.57 (0.21–1.57)	

Abbreviations: ^+^ = χ^2^; The observed genotype frequency in all SNP among individuals in the control group was in agreement with Hardy–Weinberg equilibrium (*p* > 0.05). ^&^ = logistic regression adjusted for pesticide exposure and MMSE score.

**Table 5:** Stratified association of NER gene polymorphisms with PD risk in males.

Gene (SNP)	Genotype	Controls (*n* = 73)	PD Cases (*n* = 73)	OR ^&^ (CI 95%)	*p*-Value ^+^
*ERCC1* (rs11615)	C/C	33 (45.2%)	29 (39.7%)	Ref	
	C/T	34 (46.6%)	36 (49.3%)	1.20 (0.61–2.39)	0.74
	T/T	6 (8.2%)	8 (11%)	1.52 (0.47–4.89)	
*ERCC2* (rs13181)	A/A	52 (71.2%)	49 (67.1%)	Ref	
	C/A	18 (24.7%)	21 (28.8%)	1.24 (0.59–2.60)	0.85
	C/C	3 (4.1%)	3 (4.1%)	1.06 (0.20–5.51)	
*XPA* (rs1800975)	C/C	42 (57.5%)	38 (52%)	Ref	
	C/T	20 (27.4%)	18 (24.7%)	0.99 (0.46–2.16)	0.45
	T/T	11 (15.1%)	17 (23.3%)	1.71 (0.71–4.10)	
*XPC* (rs2228001)	A/A	39 (53.4%)	36 (49.3%)	Ref	
	A/C	29 (39.7%)	22 (30.1%)	0.82 (0.40–1.68)	0.042
	C/C	5 (6.8%)	15 (20.6%)	3.25 (1.07–9.85)	
*XPF* (rs1799801)	T/T	45 (61.6%)	48 (65.8%)	Ref	
	C/T	23 (31.5%)	22 (30.1%)	0.90 (0.44–1.83)	0.73
	C/C	5 (6.8%)	3 (4.1%)	0.56 (0.13–2.49)	

Abbreviations: ^+^ = χ^2^; ^&^ = logistic regression adjusted for uric acid, pesticide exposure and MMSE score.

**Table 6:** Stratified association of NER gene polymorphisms with PD risk in females.

Gene (SNP)	Genotype	Controls (*n* = 64)	PD Cases (*n* = 64)	OR ^&^ (CI 95%)	*p*-Value ^+^
*ERCC1* (rs11615)	C/C	35 (54.7%)	30 (46.9%)	Ref	
	C/T	21 (32.8%)	30 (46.9%)	1.72 (0.80–3.69)	0.13
	T/T	8 (12.5%)	4 (6.2%)	0.47 (0.11–1.97)	
*ERCC2* (rs13181)	A/A	31 (48.4%)	39 (60.9%)	Ref	
	C/A	27 (42.2%)	24 (37.5%)	0.76 (0.35–1.62)	0.11
	C/C	6 (9.4%)	1 (1.6%)	0.14 (0.02–1.28)	
*XPA* (rs1800975)	C/C	39 (60.9%)	36 (56.2%)	Ref	
	C/T	13 (20.3%)	23 (35.9%)	1.66 (0.71–3.86)	0.072
	T/T	12 (18.8%)	5 (7.8%)	0.40 (0.12–1.27)	
*XPC* (rs2228001)	A/A	38 (59.4%)	27 (42.2%)	Ref	
	A/C	18 (28.1%)	28 (43.8%)	2.15 (0.97–4.77)	0.16
	C/C	8 (12.5%)	9 (14.1%)	1.51 (0.50–4.61)	
*XPF* (rs1799801)	T/T	32 (50%)	39 (60.9%)	Ref	
	C/T	26 (40.6%)	21 (32.8%)	0.64 (0.30–1.38)	0.41
	C/C	6 (9.4%)	4 (6.2%)	0.52 (0.13–2.09)	

Abbreviations: ^+^ = χ^2^; ^&^ = logistic regression adjusted for uric acid, pesticide exposure and MMSE score.

**Table 7:** *ERCC1*–*ERCC2* haplotype frequencies and association with PD.

*ERCC1*	*ERCC2*	Frequency	OR ^&^ (CI 95%)	*p* ^+^
C	A	0.5354		
T	A	0.2529	1.12 (0.70–1.81)	0.63
C	C	0.1489	0.81 (0.46–1.42)	0.46
T	C	0.0627	0.92 (0.39–2.16)	0.86

Abbreviations: ^+^ = χ^2^; ^&^ = logistic regression adjusted for pesticide exposure and MMSE score.

## Data Availability

The dataset supporting the findings of this study is openly available in GitHub at: https://github.com/danielramosrosales/NER-SNPs-and-Parkinson-s-Risk-A-Case-Control-Study-in-Mexican-Mestizos (accessed on 21 August 2025).

## Supplementary Materials

The following supporting information can be downloaded at: https://www.mdpi.com/article/10.3390/brainsci15091008/s1, Table S1: Genotype frequency distribution and association of *XPC* rs2228001 with pesticide exposure among PD patients.
