# Supplementary material for: Sex-Specific Association Between XPC rs2228001 Polymorphism and Parkinson’s Disease Risk in a Mexican Population: A Case–Control Study Exploring Gene–Environment Interactions
Source: Brain Sci. 2025 Sep 18;15(9):1008. doi: 10.3390/brainsci15091008 (PMC12468217; doi:10.3390/brainsci15091008)
Supplement: Supplementary file 1 [file brainsci-15-01008-s001.zip › brainsci-3861501-supplementary.pdf]

Supplementary Table 1. Genotype frequency distribution and association of *XPC* rs2228001 with pesticide exposure among PD patients.

Genotype	No pesticide exposure (n = 95)	Pesticide exposure (n = 42)	OR[&] (CI 95%)	*p*-value[+]
A/A	40 (42.1%)	23 (54.8%)	Ref	
A/C	36 (37.9%)	14 (33.3%)	0.68 (0.30–1.51)	0.31
C/C	19 (20.0%)	5 (11.9%)	0.46 (0.15–1.39)	
